# Expert panel on monitoring radiation doses from recurrent medical diagnostic procedures: Sixth Gilbert W. Beebe Webinar

**DOI:** 10.1002/acm2.70022

**Published:** 2025-02-26

**Authors:** Donald P. Frush, Armin Ansari, James A. Brink, Ourania Kosti, David B. Larson, Martha S. Linet, Mahadevappa Mahesh, Ioannis Sechopoulos, Jenia Vassileva

**Affiliations:** ^1^ Pediatric Radiology, Children's Health Center Duke University Medical Center Durham North Carolina USA; ^2^ Radiation Studies Section, Division of Environmental Health Science and Practice/NCEH Centers for Disease Control and Prevention Environmental Protection Agency Atlanta Georgia USA; ^3^ Departments of Radiology Massachusetts General Hospital Brigham and Women's Hospital, Harvard Medical School Boston Massachusetts USA; ^4^ Nuclear and Radiation Studies Board, National Academies of Sciences, Engineering, and Medicine Washington DC USA; ^5^ Department of Radiology Stanford University School of Medicine Stanford California USA; ^6^ Division of Cancer Epidemiology and Genetics National Cancer Institute Bethesda Maryland USA; ^7^ Radiological Physics Division The Russell H. Morgan Department of Radiology and Radiological Science Johns Hopkins University School of Medicine Baltimore Maryland USA; ^8^ Department of Medical Imaging Radboud University Medical Center Nijmegen The Netherlands; ^9^ Radiation Protection of Patients Unit Division of Radiation, Transport and Waste Safety International Atomic Energy Agency Vienna Austria

**Keywords:** diagnostic imaging, radiation, radiation monitoring

## Abstract

Recurrent imaging is an essential tool for patient care but with an attendant dose from radiation exposure. Recurrent imaging has been the subject of increasing scrutiny and debate based largely on the risk from increasing cumulative doses. However, the accountability for and actions with recurrent imaging as a special component in the general construct of radiation protection in medicine is unclear. This is demonstrated by the perspectives provided by the various imaging community experts. Some perspectives may be different, but many share a common ground. Understanding these various perspectives illustrates the wide‐ranging optics in considering benefits and costs in the recurrent imaging paradigm and, moreover, the value in pursuing multi‐stakeholder‐derived harmonization for recurrent imaging and radiation dose. This move towards consensus would be to the benefit of the imaging community, referrers, and other related healthcare professionals, as well as patients, their caregivers, and the public.

## 1 REVIEW

Imaging is essential for medical care. Medical imaging often involves modalities that depend on ionizing radiation, hence entailing patient radiation exposure. These modalities consist of radiography, fluoroscopy, computed tomography (CT), and nuclear medicine. Because of radiation exposure, there are potential biological risks, and therefore, decisions about imaging include consideration of both the benefits as well as the risks. Most often for each individual decision, the benefits far outweigh any potential risk. A subset of patients, however, may undergo multiple imaging examinations, both planned (e.g., on the oncologic population) as well as unplanned (e.g., in emergency settings). Because the energy deposited in the body from the radiation exposures, hereafter referred to as dose, may be associated with the risk of radiation‐induced cancer the paradigm of recurrent imaging and resultant dose and risk have prompted ongoing critical assessment by the imaging community. Specifically, there has been growing attention on the frequency of and settings in which recurrent imaging might occur in medical practice,^1^, ^2^, ^3^, ^4^, ^5^, ^6^, ^7^, ^8^, ^9^, ^10^ as well as on the influence that the increasing risk from repeated examinations, often quantified using the effective dose as a surrogate quantity, may have on decisions for further imaging for individual patients. This topic is timely and important for several reasons. In addition to some of the more broad considerations with recurrent imaging and radiation dose (Table 1), more specific issues raised regarding recurrent imaging include (1) uncertainties with dose estimates, (2) the fidelity of such in estimating the resulting cumulative risk,^11^ (3) whether the dose history should be requisite or optional at the point of care, (4) the importance of the dose history from recurrent imaging as a factor influencing decisions in the situational (e.g., acute) setting versus settings where additional imaging is expected or likely, (5) resources necessary for monitoring of individual patient dose portfolios (including assigning responsibility for this), (6) threshold for dose history from recurrent imaging to trigger any action, and (7) the role of the agency of the provider, patient/caregiver in any formula that might warrant a dose history in shared‐decision making.

**TABLE 1:** Considerations for radiation protection relevant to recurrent medical imaging exposure.

1. Diagnostic imaging is frequent with 4.2 billion examinations estimated to be performed worldwide and, in the US, medical imaging is still a major contributor to, the per capita exposure to the population.^a^
2. Radiation protection from exposure to ionizing radiation is both familiar and essential in medical practice.
3. Radiation use/exposure/dose and attendant potential risk in medical imaging continues to be a timely topic.
4. There is a growing body of evidence addressing the science of radiation exposure and biological effects, including stochastic effects from low‐level exposure.
5. There are growing calls for improved metrics for radiation exposure.
6. Recent content related to the frequency and importance of cumulative effective dose with discourse which has generated calls for clarity.
7. Dose monitoring technology is advancing.
8. Global availability is increasing for benchmarks (such as diagnostic reference levels) attesting to some of the successes of dose monitoring.
9. There are variabilities globally in regulation and guidelines as well as cultural relevance in both the medical community and the public with respect to dose monitoring.
10. It is essential to recognize that there is an implicit fiduciary responsibility to patients, their caregivers, as well as the general public to include their “voice” in shared decision‐making about whether an examination is justified (of sufficient value) or not.

^a^ United Nations Committee on the Effects of Atomic Radiation. https://www.unscear.org/docs/GAreports/2020/UNSCEAR_Report_General_Assembly_A_76_46_Part1.pdf.

Following two technical meetings in 2019 and 2020, convened by the International Atomic Energy Agency (IAEA) to discuss recurrent imaging^12^, ^13^ (discussed in more detail below), a Joint position statement and call for actions supported by nine organizations was released for strengthening radiation protection of patients undergoing recurrent radiological imaging procedures.^14^ In part, the position supported the use of past radiation exposure in decision‐making for additional imaging stating, “When a series of imaging procedures can be reasonably foreseen … insofar as possible and reasonable, clinical and radiation dose information from the patient's previous imaging procedures needs to be made available to help strengthen the appropriate decision‐making process.” This was followed by a joint statement from the American Association of Physicists in Medicine (AAPM), the American College of Radiology (ACR), and the Health Physics Society (HPS) opposing to use of prior dose information, while emphasizing the importance of considering any previously‐obtained clinical information from prior exams, when deciding on the value of an individual imaging examination, stating that decision‐making should be based on clinical need and not on prior radiation dose.^15^ The lack of consensus in the imaging community and ensuing uncertainty about what role that past radiation exposure history should be considered in ongoing medical care, contributed to a convened consultancy meeting in February 2023 of relevant organizations by the IAEA to address similarities in the two statements, identify and pursue reconciliation of differences, and offer recommendations for the approach to recurrent medical imaging and radiation exposure. The results of this meeting, including recommendations have been published.^16^

Recognizing that there are divergent opinions on the topic of recurrent imaging on the need for exposure history in decision‐making, the following material will provide perspectives on the significance in both scale and scope, as well as the role in medical decision‐making by experts in diagnostic radiology, medical physics, epidemiology, guidance, and regulation, as well as those with specialized experience in education and quality and safety. These experts participated in the 2021 Beebe Webinar,^17^ the sixth of the series focused on promoting discussions among academic and government scientists and other interested parties concerned with radiation health effects.^18^ Material in this paper is intended to represent and supplement what was discussed during the webinar.

It is important to recognize that while there are organizational representations by these individuals, opinions are those of the authorship and not necessarily representative of the respective organizations. The material is not intended to preferentially support any perspective on the significance of and approach to recurrent imaging but to inform the reader about the issues related to recurrent radiation exposure, including diverse opinions.

## 2 DISCUSSION

### 2.1 Martha S. Linet

#### 2.1.1 Epidemiology of low‐dose radiation and health risks in medically exposed populations

Epidemiology can contribute to the debate on monitoring doses in medically exposed populations by providing risk estimates, quantifying dose‐response, and identifying populations at higher risk for adverse health outcomes associated with cumulative low‐dose (defined as <100 millisieverts [mSv]) ionizing radiation exposures. The radiation effective dose equivalent designated sieverts (millisieverts for low doses) sums over all tissues allowing for differences in types of radiation (including some responsible for greater tissue damage than others) and in greater sensitivity of parts of the body to radiation than other parts. Organ/tissue doses from radiation are measured in Gray (Gy, milligray for low doses). In collaboration with medical physicists and statisticians, epidemiologists have used proxy‐monitored dose measures of patients, medical and other radiation workers to estimate cancer and other disease risks from cumulative low‐dose radiation.^19^ Data from recent^20^ and ongoing investigations will reduce the need to extrapolate risks associated with low‐dose radiation from populations exposed to higher doses.

#### 2.1.2 Cancer risks—patient exposures

Reports of skin carcinomas and leukemia within a few years after the discovery of x‐rays in 1897^19^ led to a slow reduction in patient and medical worker exposures from x‐ray procedures during subsequent decades. During the 1950s–1970s, standard prenatal pelvimetry and abdominal x‐ray imaging of pregnant women (based on maternal interviews and medical records) were associated with up to 2‐fold elevated relative risks of childhood leukemia and other childhood cancers in offspring.^19^, ^21^ These elevated risks declined after widespread replacement by ultrasonography for prenatal monitoring in the late 1970s.^21^

Standard diagnostic x‐ray procedures in childhood and adolescence have not generally been linked with increased cancer risks, but CT scans at these ages have been associated with excess relative risks per mGy of leukemia and brain tumors in dose‐response analyses using organ dose estimates in large cohorts.^19^, ^21^, ^22^, ^23^, ^24^ Risks remained significantly elevated after assessing the impact of predisposing medical or genetic disorders.^25^ Follow‐up of nine pooled cohorts of children and adolescents with <100 millisieverts (mSv) of cumulative radiation exposures revealed elevated risks of acute myeloid and acute lymphoblastic leukemia.^26^

Few epidemiologic studies have evaluated standard x‐rays or CT scans during adulthood with subsequent risk of cancer.^19^ Although statistical projections of lifetime excess cancer cases from CT scans have estimated that 1%–3% of future cancers may be associated with these exposures,^19^ these projections may be decreased by dose reduction due to technological improvements^27^ or fewer examinations, for example from changes in clinical practice, institutional policies, or insurance reimbursement. Few and relatively small studies and inconsistent results limit the interpretation of cancer risk estimates among young patients undergoing fluoroscopic (e.g., cystourethrograms or cardiac angiography) procedures.^21^ Large, cohort follow‐up studies of children and adolescents exposed to low‐dose ionizing radiation from CT scans (designated EPI‐CT including 948 000 patients from 11 countries)^23^, ^24^ and of cardiac catheterization (HARMONIC targeting 100 000 patients from seven countries)^28^ using organ dose estimates are ongoing to provide more precise estimates of cancer risks.

#### 2.1.3 Cancer, cataracts, and cardiovascular disease risks—medical radiation workers and other populations

Because medical radiation workers are required to wear dosimeters to monitor their occupational radiation exposures while patients do not, epidemiological studies of workers can provide estimates of patient risks through extrapolation and inform patient guidelines and protection measures. Medical radiation workers who first worked before 1960 experienced excess risks of leukemia, breast, and skin cancers, but those who first worked after 1960 have not consistently experienced increased cancer risks.^20^ However, a large study of U.S. radiologic technologists found small increases of several types of cancer in medical radiation workers who perform higher‐dose procedures including fluoroscopically‐guided procedures^29^ or those involving radionuclides.^30^ The study of U.S. radiologic technologists has linked cataracts with <100 mGy of lens dose radiation.^31^ A few studies of nuclear workers have shown increased risks of all circulatory, ischemic cardiac, and cerebrovascular disease at <100 mSv,^31^ but there are limited studies of these outcomes in patients with cumulative low‐dose exposure. One systematic assessment of potential biases and meta‐analysis of 26 epidemiologic studies of low‐dose radiation environmental found bias in only a few (*N* = 4), and small but statistically significant increases for low‐dose radiation and solid tumors in adults and for leukemia in children and adults after excluding the studies with bias.^32^

#### 2.1.4 Monitoring diagnostic imaging radiation exposures: An epidemiologist's perspective

Based on epidemiologic data, population subgroups to consider as high value for monitoring for future serious health outcomes (e.g., radiogenic cancers, cataracts, cardiovascular diseases) are neonates, children, adolescents, and young adults who have undergone repeated higher‐dose imaging procedures. Such procedures would include fluoroscopic cystourethrograms, fluoroscopically‐guided cardiac examinations, certain procedures involving radionuclides (e.g., positron emission tomography (PET)/CT), and CT examinations (e.g., for monitoring of cancer, autoimmune, other chronic medical conditions, genetic or congenital disorders, and for diagnostic evaluation of those whose sports activities or work are likely to involve repeated head injuries). Interpretation of epidemiologic studies must consider the lack of or imperfect individual dose estimates and small sample size, particularly for rare cancers. Other shortcomings may include selection and other biases, lack of information about potential confounders, and incomplete ascertainment of all outcomes.

#### 2.1.5 Reducing cumulative radiation exposures

Dose monitoring is a valuable tool for reducing cumulative radiation exposures, but more efforts are needed. For example, improved education and training of medical students and physicians‐in‐training on principles of radiation science, the concepts of justification and optimization, introduction to best practices and protocols for diagnostic imaging used in initial patient work‐up and in long‐term follow‐up, and working collaboratively with medical physicists and radiologic technologists to optimize radiation exposures should reduce the numbers of clinically unnecessary imaging examinations and optimize dose levels. The key goals of such education and training are to improve clinical decision‐making by maximizing consideration of the benefit‐risk ratio in clinical practice, optimizing practices and protocols, decreasing unnecessary imaging examinations and radiation exposures, and increasing the use of modalities (ultrasonography and magnetic resonance imaging) that do not expose patients to ionizing radiation. Simulation studies could quantify the reduction in cancer and other serious radiation‐related disease risks identified in epidemiologic studies if unnecessarily repetitive diagnostic imaging examinations were reduced.^33^

### 2.2 Jenia Vassileva

#### 2.2.1 Tracking radiation exposures from medical diagnostic procedures: IAEA perspective

The International Basic Safety Standards (BSS)^34^ states that dose limits do not apply to medical exposure of patients, and thus, efforts are towards improved justification and optimization of radiation protection. For the justification of medical exposure for an individual patient, the BSS requires this to be “ …carried out by means of consultation between the radiological medical practitioner and the referring medical practitioner, as appropriate, with account taken, in particular for patients who are pregnant or breast‐feeding or are pediatric, of: (a) the appropriateness of the request; (b) the urgency of the radiological procedure; (c) the characteristics of the medical exposure; (d) the characteristics of the individual patient; and (e) relevant information from the patient's previous radiological procedures.” This is further detailed in the Safety Guide on Radiation Protection and Safety in Medical Uses of Ionizing Radiation,^35^ specifying that “the results (images and reports) of previous examinations should be made available, not only at a given radiology facility but also for consultation at different facilities,” and that “individual patient exposure records should be used to facilitate the decision‐making process.” For the optimization, which aims at keeping the exposure of patients to the minimum necessary to achieve the required diagnostic or interventional objective, tools to be used include diagnostic reference levels (DRLs), local dose assessments for benchmarking with DRLs, and optimization review. The Safety Guide also specifies that the justification and optimization process can be facilitated using digital hospital information systems and their integration.

The new IAEA Safety Report on Patient Radiation Exposure Monitoring in Medical Imaging details the process of recording and collecting exposure data with a focus on automatic radiation exposure monitoring (REM) systems and their various analytical uses.^36^ In addition to ensuring optimized and consistent imaging practice of modality‐specific dose and image quality metrics, monitoring can support safe and precise imaging of individual patients supported by the monitoring of exposure data for an individual patient over time. Such a methodology for exposure monitoring of individual patients was proposed within the IAEA Smart Card Project^37^ and the Joint Position Statement by seven organizations^38^ suggesting that countries need to work for achieving increased coverage of both local and global patient REM.

An example of a benefit of an automatic REM system is found from population‐based studies with large cohorts. A conservative extrapolation was made that around 1 million patients who undergo multiple CT examinations over a short period accumulate a dose of ≥100 mSv.^1^, ^6^, ^9^ Such studies triggered an international discussion that motivated the IAEA to call Technical Meetings in 2019 and 2020 with the participation of the IAEA Member States, international organization and professional bodies representing perspectives of research, clinical practice, industry, and regulatory agencies. The conclusions of this consultation were reflected in the Joint Position Statement and Call for Action by nine organizations published in 2021.^14^ The Statement identified nine actions to respond to this new issue, that include an additional focus on appropriateness and imaging strategies for clinical conditions where recurrent imaging is likely to lead to increased radiation exposure, further dose reduction through technological development, optimization focused on imaging protocols for clinical problems involving multiple imaging, strengthening education and training and strengthening communication. A specific action calls for monitoring the radiation exposure history of patients, as a part of the automatic REM systems to provide for effective monitoring over time of individual patients in more generic metrics such as type of radiological procedure, estimated effective dose or patient‐specific organ dose estimates.

**FIGURE 1:**
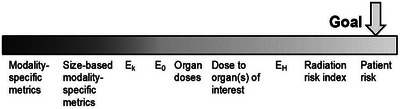
Spectrum of dose metrics representing patient risk. Patient exposure metrics ranging from modality‐specific (left) to patient‐oriented (right). E_k_ is effective dose and is calculated for a generic reference person from modality‐specific standard conversion factors; E_0_ uses organ doses calculated for a generic reference person; E_H_ employs organ doses calculated derived from the anatomical definition of the actual patient (with permission; International Atomic Energy Agency, Patient Radiation Exposure Monitoring in Medical Imaging, Safety Reports Series; 2023, No. 112, IAEA, Vienna).

The IAEA Safety Report and the Position Statement address two questions related to monitoring that lack consensus. The first is what to monitor. Monitoring for individual patients can be applied to procedures, listing various radiological examinations an individual patient has undergone, such as through the electronic health record or the estimated patient dose associated with the examinations. Ideally, a relevant dose quantity representing individual patient risk is required (Figure 1). For an imaging procedure with potential for tissue reactions, knowledge of cumulative dose to the skin from previous procedures can be a factor when planning the proper timing of the next procedure and its optimization, as well as for the patient's follow‐up for potential tissue reactions. For a procedure with the potential for stochastic effects, justification for the examination needs to be primarily based on a benefit‐risk consideration for that particular examination. Even so, the patient's prior radiation history in terms of patient‐oriented risk metrics would additionally inform the overall benefit‐risk analysis. Such metrics include organ dose or patient‐specific effective dose.^36^ Further standardization is needed of the methods for these estimates with accounting for the associated uncertainties.

The second question is would there be misinterpretation and misuse of radiation exposure history data by referring physicians and patients. The conclusions drawn from recent surveys among patients and referring physicians from different countries indicate a prevailing opinion that knowing radiation exposure history of the patient would inform decision‐making.^39^, ^40^ One consideration for radiation exposure history would be the resources needed for monitoring, the value of which depends on the type of the healthcare system and the strength of the radiation protection systems.^41^ The IAEA report recommends that the user groups of REM systems and their access level should be carefully considered to ensure proper use, with an emphasis on their education and training.^36^ A recent publication from Europe^2^ proposed actions for reducing the number of recurrent imaging examinations, which is in line with the Joint Position Statement^14^ and the IAEA Safety Report^36^ advising that REM systems, designed to meet local, regional or national needs and standards, have to be disseminated as well as integrated with other healthcare IT systems including EHRs.

An additional important argument in favor of radiation exposure history monitoring is that the improved access to patient‐specific organ doses, linked to patient's health records, could allow for strengthening the studies of low‐dose effects from medical exposure, particularly in childhood, while accounting for reverse causation and confounding factors.

The Joint Position Statement initiated by the IAEA was intended to prompt greater dialogue with a focus on patient safety. There is a need for further research and wider consultation to better integrate radiation protection and clinical perspectives and to find tools to support the risk‐to‐benefit analysis that accounts in holistic for all incidental and long‐term benefits and risks for patients, their clinical history, and specific needs.

### 2.3 Armin Ansari

Assessment of patient medical exposures is of great interest at national and international levels. At the national level, the National Council on Radiation Protection and Measurements (NCRP) has provided the latest assessment of medical exposures and detailed information on patient diagnostic and interventional radiation exposures for the U.S. population.^27^ The United Nations Scientific Committee on Effects of Atomic Radiation (UNSCEAR) recently released their latest assessment of medical exposure of patients for the global population.^42^ As both reports illustrate, medical exposure of patients remains by far the largest human‐made source of radiation exposure of the population. These national or global assessments of parameters such as frequency of medical examinations and procedures involving the use of ionizing radiation, as well as associated doses per procedure, can inform the development of policies and strategies for the optimization of healthcare delivery and improve practice. Similarly, laudable initiatives such as the American College of Radiology (ACR) Dose Index Registry (DIR) and available benchmarks for various procedures are invaluable for the promotion of best practices among medical facilities that use ionizing radiation. While the ultimate beneficiaries of these improvements are the patients, these efforts fall short of meeting the patient's needs for information.

As stated in the Patient Right to Know Act of 1996 [H.R. 2976],^43^ “Patients cannot make appropriate healthcare decisions without access to all relevant information relating to those decisions.” In 2004, President George W. Bush unveiled his Health Information Technology Plan, issued Executive Order 13335, and charged the Office of National Coordinator with the critical responsibility of ensuring that every American had access to his or her electronic health information and establishing connectivity of health information technology. In 2009, the Health Information Technology for Economic and Clinical Health (HITECH) Act, enacted as part of the American Recovery and Reinvestment Act of 2009, was passed by U.S. Congress, and signed into law to encourage the adoption and meaningful use of health information technology.^43^ Its purpose is to promote nationwide, standards‐based health information exchange and to advance person‐centered and self‐managed health.

The radiation dose that patients receive from a medical procedure (regardless of what metric or dosimetric quantity is used for comparison with established benchmarks) needs to be regarded as a relevant component of the patient's health record and provided to the patients as they exercise their rightful choice in using providers for specific procedures. Monitoring and reporting of individual patient's doses (regardless of whichever metric is used) provides patients with the knowledge that there is accountability/responsibility in the delivery of medical radiation and improves their confidence in that healthcare providers’ care.^38^ A corollary benefit to monitoring individual medical doses is the opportunity it provides to examine and help address any disparity regarding access to and provision of diagnostic and therapeutic radiation medical services. Associations with racial, ethnic, and socioeconomic disparities in provision and quality of healthcare, in general, have been recognized by several organizations.^44^, ^45^, ^46^ The ACR previously developed a Health Equity Coalition to “explore steps radiology professionals may take to drive improved outcomes for those impacted by regional, racial and economic‐related healthcare disparities.” Monitoring of individual patient doses can potentially contribute to those efforts.

One barrier often mentioned in monitoring of individual patient doses is the uncertainty as to which dose metric needs to be included or tracked. From the patients’ perspective, it is reasonable that whichever metric is used it needs to include a benchmark for comparison. This is similar, for example, to a variety of other analytical parameters reported in a patient's blood test where patients may not necessarily understand its value without the range of normal values.

Another barrier often mentioned in monitoring of individual patient doses is the lack of a unique patient identifier which can be used to integrate medical records across various care providers. It is noteworthy that such a unique identification system already exists for Medicare beneficiaries in the United States, and the same or similar system can be used for the population aged 64 years or younger. Therefore, these and other barriers often cited are not insurmountable. Information technology solutions either already exist or can be developed.

### 2.4 Ioannis Sechopoulos

Monitoring cumulative exposures across medical diagnostic procedures at the individual patient level supposes that there would be some benefit to doing so. However, there is no clear benefit to such an undertaking, and this could bring about multiple negative consequences for the care of our patients.

Providing optimal care for our patients involves a team effort. On the one hand, the team of clinicians involved in a patient's care, including the referring physician and the radiologists, need to perform only the necessary and most appropriate diagnostic procedure(s). Which imaging procedure is appropriate depends on many factors, many of which are, for example, used as guidance in standard documents such as the American College of Radiology Appropriateness Criteria.^47^ Another factor that physicians need to consider is what prior imaging procedures, if any, the patient has already undergone. This prior history is important, however, not due to the prior exposures that the patient could have experienced, but due to the possible clinical information, or lack thereof, already gleaned from those examinations.

On the other hand, radiographers/technologists, imaging physicians including radiologists, and medical physicists need to work together to *optimize* the radiation dose used during the acquisition of *each* and *every* diagnostic procedure involving ionizing radiation. An image acquisition that uses more *or less* than that optimized dose is inappropriate. Therefore, the entire healthcare‐providing team works together to ensure the most appropriate diagnostic procedure is performed, and that it is performed in the most optimal way possible. As mentioned, however, the history of prior doses or the total accumulated dose that the patient received does not affect, in any way, the decision for the type and performance for an individual examination.

#### 2.4.1 Why does prior accumulated dose have no effect in determining the most appropriate next diagnostic procedure in a situational setting?

There are two issues that are relevant in this discussion, and many times, these are erroneously brought up as a single one. First, do the diagnostic‐level doses of radiation increase the risk of developing cancer in the future? Second, if there is such an increase in risk, does that increase become larger after being exposed to prior doses? These are clearly two distinct questions that need to be addressed separately.

#### 2.4.2 Do diagnostic‐level doses of radiation increase the risk of developing cancer in the future?

Diagnostic imaging procedures use dose levels that are below those that are known to increase the risk of future cancers. This does not mean that diagnostic imaging dose levels do not increase that risk, but that there is still debate that this is the case. As the epidemiology section of this article states, there are conflicting results,^20^ and more studies that do not involve the extrapolation down from very high doses are needed.^20^, ^48^ Some more recent studies seem to indicate that there may be a correlation between increased risk of cancer and increased imaging, but these involve diseased populations, and therefore, there might be other factors involved*. If, however, there is indeed an increase in risk, this increase is far outweighed by the benefit of performing an appropriate imaging procedure*. Therefore, medical imaging should always be performed only when it is clinically appropriate and under optimized conditions, as described above. Not performing an appropriate diagnostic procedure, or not performing it with the most appropriate imaging modality or technique for the clinical question at hand, may adversely impact patient care by decreasing the diagnostic yield (including confidence).

#### 2.4.3 If there is an increase in cancer risk due to diagnostic exposures, does that increase become larger after being exposed to prior doses?

Currently, the most conservative model relating radiation dose to risk is that used for radiation protection purposes, that is when considering personnel being exposed due to their work. In this case, the worker being exposed draws no benefit from this exposure, so using the most conservative model for this scenario is appropriate. This is, of course, different than in the case of a patient being exposed during a medical imaging scan; the patient benefits from undergoing an appropriate and optimized diagnostic procedure. For radiation protection purposes, the currently accepted radiation dose‐risk model is the linear no‐threshold (LNT) model. The no‐threshold component means that exposure to any amount of radiation, no matter how small, even a single x‐ray, increases the risk of future cancer development to some degree. The linear aspect means that the increase in risk corresponds with the increase in exposure in a constant fashion. That is, if, for example, a dose of X increases the risk of future cancer development by Y%, then a dose of 2× increases that risk by 2Y%. According to the LNT model, this relationship does not change, and there is no evidence that this relationship changes due to prior exposures. Therefore, the dose‐related risk aspect of the first, second, third, or tenth diagnostic imaging procedure remains the same. Hence, if the first CT scan of a patient was appropriate because the benefit‐risk ratio was positive, then the fifth and the tenth CT, as long as they are still clinically appropriate, result in the same positive benefit‐risk ratio.

Finally, when considering tissue reactions caused at much higher radiation doses than those involved in diagnostic imaging procedures, such as skin erythema/burns, epilation, or cataracts, we have a clear understanding of the radiobiological mechanisms that cause a follow‐up exposure to cause more damage after the first one. However, there are no known such mechanisms that would explain why the stochastic risk due to a certain low‐dose exposure today is higher due to prior low‐dose exposures.

#### 2.4.4 What is the harm in monitoring each patient's exposures, even if not really relevant?

First, performing such monitoring appropriately would involve resources. In addition, exposures to different parts of the body from different imaging modalities are not easily converted to the same single dose metric. How does the absorbed dose to the brain and other organs in the head from a head CT compare, and is possible to combine with a whole‐body exposure due to injection of a radiopharmaceutical tracer during a PET scan? Applying the concept of effective dose at the individual patient level is not appropriate. How much effort would it take to get an accurate, patient‐specific, absorbed dose estimate from a multiple‐view fluoroscopic procedure? Obtaining patient‐specific dose estimates from diagnostic procedures is a topic of much research, but still with many limitations for most diagnostic imaging modalities.

More importantly, if the electronic medical records of patients monitor these cumulative dose values, they are fraught with danger of being misused. Clinicians with an incomplete or incorrect understanding may erroneously opt for modalities that do not involve ionizing radiation only due to these dose records, even if clinically less appropriate than other modalities. It has been shown that even physicians who do have the appropriate understanding of these concepts inappropriately change the imaging recommendation due to being exposed to the dose history of the patient.^49^ Insurance companies may also use these records to justify limiting some more expensive, but more appropriate, imaging modalities for more affordable ultrasound, even if not clinically appropriate. Finally, patients might be concerned about the meaning of their *dose history*, and be at the least anxious, but also reluctant or refuse to undergo the next CT scan that can have a significant benefit in management, even when normal.

There are appropriate reasons to gather records of patient exposure from diagnostic procedures, but these do not require the records to be tied to each patient's medical record. The main reasons are quality control of imaging systems and optimization of protocols, especially when the data is detailed enough to allow for evaluation and optimization of protocols of patients of different characteristics, mainly in terms of size and clinical indication for the imaging procedure. However, the information needed for these endeavors need not be displayed in each individual patient's medical record including being present when an individual examination is being considered, but rather in separate databases to be used by the healthcare team to optimize its devices, protocols, and procedures.

All these factors have led the AAPM, ACR, and HPS, eventually endorsed by several other societies to issue the Position Statement, that reads, in part, that “…the decision to perform a medical imaging examination should be based on clinical grounds, including the information available from prior imaging results, and not on the dose from prior imaging‐related radiation exposures….”.^15^

### 2.5 David B. Larson

In the spirit of “you can't improve what you don't measure,” at first blush, it might seem logical that radiation dose tracking would help drive down inappropriate radiation exposure. However, the reality is that radiation dose tracking provides little useful information that impacts how much radiation is used in medical imaging.

First, let us consider how a radiation tracking system might affect the performance of unnecessary examinations. An examination is warranted if the likely benefit of the new information provided by the imaging outweighs the costs and risks in terms of potential adverse effects of ionizing radiation. The benefit of the new information is related to its ability to help guide patient management in the specific situation in which uncertainty exists. If the likely benefit of the new information outweighs the costs and risks, then the examination is warranted, regardless of what has happened in the past. In other words, information regarding past radiation exposure has no legitimate bearing on the decision of whether to proceed with a given imaging examination. The intuitive but misguided belief that patients with an extensive history of radiation exposure should be treated differently than those with more limited radiation exposure reflects a common cognitive bias, akin to the “sunk cost fallacy,” in which the decision to continue with an effort is inappropriately biased by past expenditures rather than evaluating the decision at hand on its own merits, independent of past decisions.

One might imagine that a tracking system might be helpful in that it could bring to an ordering clinician's awareness of multiple prior imaging examinations, which might change the clinician's propensity to order a given imaging examination. However, while such a system might indeed be helpful in the setting where a referring provider does not have access to prior imaging history, it is not the use of radiation that affects the decision, but rather it is the knowledge of the existence of prior imaging that might obviate the need for additional imaging. This is not a problem of dose management but rather one of information management. For this application, instead of developing a system to track radiation dose, efforts should be focused on enabling clinicians’ access to information about the patient's prior imaging.

While there is no direct benefit of a dose‐tracking system, there is, in fact, a risk, in that the presence of a dose‐tracking system highlighting prior radiation dose exposure reinforces the cognitive bias of inappropriately incorporating prior radiation dose into the ordering decision, thus embedding the sunk cost fallacy into the healthcare infrastructure.

Second is the question of how a radiation dose‐tracking system might affect the amount of radiation used when performing a given examination. The ALARA principle calls for the use of the minimum amount of radiation necessary to provide the information needed, meaning enough radiation to answer the clinical question and no more, in every examination. In other words, the amount of radiation to be used in any given study is independent of past radiation exposure. Thus, a radiation history provides no benefit regarding the radiation dose used in a given study and could perpetuate the misguided belief that a lower dose should be used in a patient with a history of radiation exposure.

Additionally, the feasibility of establishing a comprehensive dose‐tracking system and providing past dose history to all clinicians at the time of order for all relevant imaging is questionable. Enabling information to cross organizational boundaries is particularly challenging. This requires the establishment of a universal patient identifier (or other mechanism of patient identification), agreement upon technical definitions and standards, granting of data access permissions, assurance of data security, establishment of technical infrastructure, and ongoing system oversight, support, and quality control. This is even further exacerbated when considering difficulties in crossing jurisdictional boundaries and translating between languages. To be effective on a global scale, such a system would need to serve billions of patients and hundreds of thousands of care providers over decades. It is hard to overstate the expense of such an endeavor. The political feasibility of such a system is perhaps even more formidable, especially given the fact that political support from the expert medical community is mixed at best.

Even if such a tracking system could be widely implemented, it would not directly reduce a patient's dose. Dose optimization is the product of coordinated efforts by radiologists and other imaging professionals, medical physicists, and technologists/radiographers to carefully manage protocols and examination performance within a given organization. Organizations must make an investment of time and resources for this purpose. Accountability for activities resulting in consistent dose optimization happens at the institutional level, which generally does not follow patients longitudinally as they move between practices/institutions and healthcare systems.

This raises the point of the difference between tracking and monitoring. A monitoring system implies a program accompanying a dose‐tracking system that includes oversight and enforcement. Given the impracticality of developing even a tracking system, how one would practically create a comprehensive monitoring program that includes these components is difficult even to conceive.

In short, if the global community is to spend its effort and attention to decrease unnecessary radiation dose, (which it should), those resources should be used to facilitate and incentivize local institutions to develop the requisite internal programs that ensure doses are as low as reasonably achievable for all patients.

## 3 SUMMARY

Recurrent imaging is an essential tool for patient care, but with an attendant dose from radiation exposure. Recurrent imaging has been the subject of increasing scrutiny and debate based largely on the risk from repeated doses. However, the accountability for and actions with recurrent imaging as a special component in the general construct of radiation protection in medicine is unclear. This is demonstrated by perspectives, some different but many of which find common ground, provided by the various imaging community experts. Understanding these various perspectives illustrates the wide‐ranging optics in considering benefits and costs in the recurrent imaging paradigm and, moreover, the value in pursuing multi‐stakeholder‐derived harmonization for recurrent imaging and radiation dose. This move towards consensus would be to the benefit of the imaging community, referrers, and other related healthcare professionals, as well as for patients, their caregivers, and the public.

## AUTHOR CONTRIBUTIONS

Each author has met all of the following criteria: Substantial contributions to the conception or design of the work; or the acquisition, analysis, or interpretation of data for the work; drafting the work or revising it critically for important intellectual content; final approval of the version to be published; and agreement to be accountable for all aspects of the work in ensuring that questions related to the accuracy or integrity of any part of the work are appropriately investigated and resolved. Each author has made substantial contributions to the drafting and revision of the work, approved the submitted version, and agreed both to be personally accountable for the author's own contributions and to ensure that questions related to the accuracy or integrity of any part of the work, even ones in which the author was not personally involved, are appropriately investigated, resolved, and the resolution documented in the literature.

## CONFLICT OF INTEREST STATEMENT

Donald P. Frush, Armin Ansari, Ourania Kosti, Martha S. Linet, Mahadevappa Mahesh, and Jenia Vassileva: Declare no conflicts of interest. James A. Brink: Board of Directors, Accumen, Inc. David B. Larson: Research funding from the Gordon and Betty Moore Foundation; shareholder for Bunker Hill Health. Ioannis Sechopoulos: Research agreements with Siemens Healthcare, Canon Medical Systems, ScreenPoint Medical, Sectra Benelux, Volpara Healthcare, Lunit, and E‐COM; a speaker agreement with Canon Medical, and is a Scientific Advisory Board member of Koning Corp.
